# Advances in the cellular immunological pathogenesis of type 1 diabetes

**DOI:** 10.1111/jcmm.12270

**Published:** 2014-03-14

**Authors:** Min Li, Lu-Jun Song, Xin-Yu Qin

**Affiliations:** a Department of General Surgery, Zhongshan Hospital, Fudan UniversityShanghai, China

**Keywords:** type 1 diabetes, autoimmune disease, T lymphocyte, islet cells, immunological mechanism

## Abstract

Type 1 diabetes is an autoimmune disease caused by the immune-mediated destruction of insulin-producing pancreatic β cells. In recent years, the incidence of type 1 diabetes continues to increase. It is supposed that genetic, environmental and immune factors participate in the damage of pancreatic β cells. Both the immune regulation and the immune response are involved in the pathogenesis of type 1 diabetes, in which cellular immunity plays a significant role. For the infiltration of CD4^+^ and CD8^+^ T lymphocyte, B lymphocytes, natural killer cells, dendritic cells and other immune cells take part in the damage of pancreatic β cells, which ultimately lead to type 1 diabetes. This review outlines the cellular immunological mechanism of type 1 diabetes, with a particular emphasis to T lymphocyte and natural killer cells, and provides the effective immune therapy in T1D, which is approached at three stages. However, future studies will be directed at searching for an effective, safe and long-lasting strategy to enhance the regulation of a diabetogenic immune system with limited toxicity and without global immunosuppression.

- Introduction
- Islet autoantigen
  – Insulin
  – GAD
  – IA-2
  – ZnT8
- Immune cells
- T lymphocytes in the pathogenesis of T1D
- CD4+ T lymphocytes and T1D
  – Th1 cells
  – Th2 cells
  – Th17 cells
  – Tregs
- CD8+ T lymphocytes and T1D
- B lymphocytes in the pathogenesis of T1D
- NK cells in the pathogenesis of T1D
- APC in the pathogenesis of T1D
- Other innate immune cells
- Conclusion

## Introduction

Type 1 diabetes (T1D) is an autoimmune disease whereby antigen-specific T cells selectively destroy insulin-producing pancreatic β cells [1–3]. A dramatic increase in T1D incidence was recorded in most developed countries in the past 40 years [2]. It is a polygenic disorder where loci within the human leucocyte antigen (HLA) account for most of genetic susceptibility. Non-genetic factors, most likely environmental, are also involved in the pathogenesis of the disease resulting in a T cell-mediated autoimmune attack against pancreatic β cells [4]. In 1965, Gepts *et al*. first identified inflammatory infiltrates in pancreatic islets, which have since then become a hallmark of T1D termed ‘insulitis’ [5,6]. Current evidence suggests that initiation of T1D requires both CD4^+^ and CD8^+^ T cells; that autoreactive T cells differentiate into effectors by engaging β-cell antigens on local antigen-presenting cells (APCs); that initiating CD4^+^ T cells are insulin reactive; and that CD8^+^ T cells play a major role as β-cell killers [7]. T cells can directly kill β cells *via* cell-to-cell contact, through a cytotoxic process, but they can also influence their destruction through other factors, including the release of pro-inflammatory cytokines, granzyme B, or perforin, and possibly signalling through pathways of programmed cell death [8]. A significant number of other immune cell types including B cells, NK cells, natural killer T cell (NKT), γδT and macrophages have been implicated in T1D progression. Although the precise sequence of events remains ill defined, recent studies have brought forth a renewed understanding of cellular immunological mechanism.

## Islet autoantigen

**Fig. 1:**
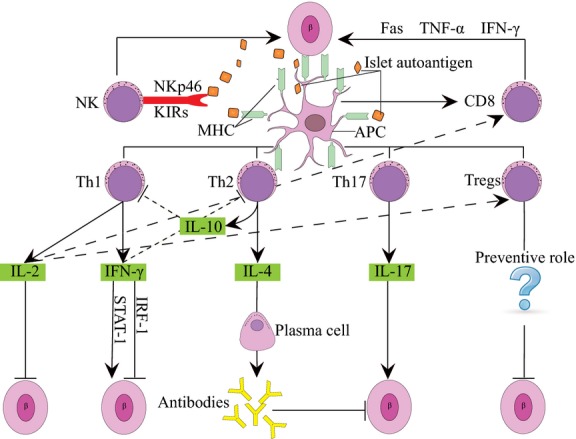
β-cells are damaged by various factors and the released autoantigens are presented by antigen-presenting cells. Then CD4+ T, CD8+ T and NK cells are activated, and CD4+ helper T lymphocytes differentiate into Th1, Th2, Th17 and Tregs. Th1 cells can destroy the islet β cells and accelerate the course of T1DM *via* production of IL-2 and IFN-γ. IL-2 has been shown to prevent diabetes, while it can activate CD8+ T cells and Tregs. In addition, IFN-γ plays a dual role in the destruction of β cells *via* the signal transducer and activator of transcription-1 (STAT-1) pathway and in protection *via* the IRF-1 pathway. Th2 cells mainly produce IL-4 and IL-10, which are responsible for strong antibody production, have been ascribed with a protective role. Th17 can destroy the islet β cells by secreting IL-17. Whether Tregs play a preventive role in the pathogenesis of T1DM remains a question. In addition, NK cells are involved in direct killing of β cells through the interaction of NK cell markers, such as NKp46 and KIRs. Furthermore, CD8+ T cells contribute to the development of T1DM by secreting proteins such as Fas, and cytokines such as TNF-α and IFN-γ.

The identification of islet autoantibodies has important implications in the diagnosis and prediction of T1D. Autoantibodies directed against islet autoantigens such as insulin, glutamic acid decarboxylase 65 (GAD 65), islet antigen-2 (IA-2) and Zinc transporter 8 (ZnT8) have been demonstrated to be markers of the islet autoimmunity that precede clinical onset of T1D [9,10] (Fig. 1).

### Insulin

Insulin is a critical autoantigen specifically expressed on the β-islet cells, which is perceived as the target antigen to cause autoimmune diabetes for a long time [11]. It has been reported that insulin peptide A:1-12 and B:9-23 might be essential targets of the immune destruction for human and non-obese diabetic (NOD) mouse respectively [12–14]. Studies of multiple countries have reported that insulin autoantibody (IAA) takes an important role in diabetes prediction [15]. In man, IAA was frequently present as early as 9 months of age [15]. Non-obese diabetic mice had high levels of IAA at 8 weeks of age, which strongly correlated with early development of diabetes, and, in a similar manner, children persistently expressing IAA early in life progressed to diabetes much earlier [15]. In addition, recent experiments have shown that mucosal administration of insulin or gene disruption of insulin prevent the onset of diabetes in the NOD model of diabetes [11,16].

### GAD

The enzyme GAD is of great importance for the neurotransmission in the central nervous system and for treatment of pain and neurological disease, which is also released in pancreas [17]. GAD exists in two isoforms, GAD-65 and GAD-67, which are the products of two different genes and differ substantially only at their N-terminal regions [18]. Only GAD65 is expressed in the β cells of human islets, the autoantibody response is primarily to this isoform, and GAD67 antibodies add little to the detection of T1D [19]. Autoantibodies to GAD65 are observed months to years before the clinical onset of diabetes and are present in the sera of 70–80% of patients with T1D [20–22]. A few earlier reports indicate that treatment using GAD 65 formulated with aluminium hydroxide (GAD-alum) have significant beneficial effects on T1D, however, in the latest trials, treatment with GAD-alum did not significantly improve clinical outcome. [23–25].

### IA-2

IA-2 and its paralog, IA-2 β, are major autoantigen found after GAD in T1D, which are transmembrane protein-tyrosine phosphatase-like proteins belonging to an evolutionarily conserved family [26]. IA-2 β is similar in many respects to IA-2, especially in its intracellular domain, which is 74% identical to IA-2 [27]. IA-2-deficient (IA-2^−/−^) mice showed impaired insulin secretion after intraperitoneal injection of glucose as well as elevated glucose level in a glucose tolerance test [28]. It is estimated that about 65% (range 55 ± 75%) of newly diagnosed type 1 diabetic patients have autoantibodies to IA-2 and between 35% and 50% of type 1 diabetic patients have autoantibodies to IA-2 β [27]. In particular, novel autoantibodies, such as those against the initial 277 amino acid residues of extracellular domain of the neuroendocrine antigen IA-2, had a predictive rate of 100% in a 10-year follow-up [8].

### ZnT8

ZnT8 is an islet β-cell secretory granule membrane protein recently identified as an autoantibody antigen in T1D [29–31], which is highly β-cell specific unlike GAD and IA-2. ZnT8 contains six transmembrane domains and a histidine-rich loop between transmembrane domains IV and V, like the other ZnT proteins [32]. A high-ranking candidate, the ZnT8 was targeted by autoantibodies in 60–80% of new-onset T1D compared with <2% of controls [33]. ZnT8_107–115_, ZnT8_115–123_ and ZnT8_145–153_ derived from ZnT8 might be capable of inducing specific CTLs and played a vital role in T1D [34]. Vaziri *et al*. have further reported that the assay of ZnT8-TripleA would be more suitable to analyse patients with newly diagnosed diabetes as this assay demonstrated high sensitivity and very high specificity [9]. Studies in humans have shown that reagents that target ZnT8-specific T cells could have therapeutic potential in preventing or arresting the progression of this disease [30,35]. Unlike GAD and IA2, ZnT8 is highly β-cell specific, and thus, ZnT8 antibodies measurements may be useful in monitoring islet destruction after onset and in evaluating therapeutic interventions that limit β-cell-specific autoreactivity or restore β-cell mass [33].

## Immune cells

Many cell subsets such as T cells, B cells, NK cells, APCs and other innate immune cells participate in the damage of pancreatic β cells, which ultimately lead to T1D. In the next section, we detail current information regarding different immune cells and pathogenesis of T1D.

## T lymphocytes in the pathogenesis of T1D

T lymphocytes* crucial role in the autoimmune process leading to T1D is considered to be the final executors of β-cell destruction. This is demonstrated by the precipitation or prevention of diabetes by transfer or elimination of CD4^+^ or CD8^+^ T cells respectively [6]. T cells can be divided into CD4^+^ T cells and CD8^+^ T cells according to the expression of surface CD molecule; here, we will discuss the relationship between different T cell subsets with T1D respectively.

## CD4^+^ T lymphocytes and T1D

CD4^+^ T lymphocytes are mainly involved in cellular immune response, and play important roles in the activation and proliferation of CD8^+^ T lymphocytes and B cells. Human CD4^+^ cells from the pancreatic lymph nodes of patients with T1D respond to the first 15 amino acids of the insulin A-chain [36]. Insulin-autoreactive CD4^+^ T cells have also been described in T1D patients, and there is evidence suggesting that high-avidity insulin-reactive thymocytes may evade central tolerance in such patients [7].

CD4^+^ T cells can be divided into type 1 T helper (Th1), Th2, Th17, regulatory T cells (Tregs) and so on according to their secretion of cytokines. Recently, there have been considerable insights into the effects of Tregs in the pathogenesis of T1D, which have evoked great interest. It is essential to understand more clearly the role of each CD4+ T cell subset in the protection or exacerbation of various pathologies in T1D.

### Th1 cells

Th1 cells are responsible for cell-mediated immunity and phagocyte-dependent protective responses, which can also destroy the islet β cells and accelerate the course of T1D *via* production of interferon (IFN) -γ and interleukin (IL)-2. However, the role of these cytokines in the pathogenesis of T1D is complex. For example, IFN-γ plays a dual role in destruction of β cells *via* the signal transducer and activator of transcription-1 (STAT-1) pathway and in protection *via* the interferon regulatory factor-1 (IRF-1) pathway [37]. And IL-2 may have therapeutic efficacy in T1D by promoting the survival and function of Tregs, which we will describe in detail in the related part.

### Th2 cells

Th2 cells mainly produce IL-4 and IL-10, which are responsible for strong antibody production, eosinophil activation and inhibition of several macrophage functions [38,39]. Immunotherapeutic approaches like anti-CD28 stimulation, which promote and enhance the function of intraislet Th2 cells and secretion of IL-4 by these cells can effectively prevent the onset of T1D [40]. Transgenic NOD mice expressing IL-4 in the pancreatic islets are protected from the development of diabetes [41]. The onset of hyperglycaemia in NOD mice has reduced after regulated delivery of IL-4 to pancreatic β cells *in vivo* using an adenoassociated vector expressing IL-4 under the control of the mouse insulin promoter [41]. Similarly, IL-10 is an immunoregulatory cytokine that has multifunctional effects. Several lines of evidence suggested that IL-10 was important in establishing immune tolerance in NOD mice whereas other reports demonstrated that IL-10 displayed an opposite function [42]. There is now widespread recognition that Th1 cells regulate cellular immunity, whereas Th2 cells mediate humoural immunity and allergic responses [43]. Th1/Th2-cell subsets have been extensively studied, Th1 cytokines are generally believed to exacerbate, while Th2 cytokines protect from, T1D. However, more and more studies indicated that both Th1 and Th2 cytokines appear to cooperate in driving β-islet-cell destruction, eventually leading to hyperglycaemia [44].

### Th17 cells

Th17 are a subset of T helper cells producing IL-17, which are distinct from Th1 and Th2 cells. Th17 play a key role in a variety of infectious diseases, cancer occurrence and many autoimmune diseases, such as T1D, rheumatoid arthritis, multiple sclerosis and systemic lupus erythematosus [45–47]. One report demonstrated that in T1D, Th17 might induce local inflammation, which in turn might hasten the development of diabetic complications [48]. Increasing evidence has shown that therapeutic agents targeting the IL-17 molecule or directly inhibiting IL-17-producing cells regulate autoimmune diabetes, suggesting that IL-17 is involved in the pathogenesis of T1D [49]. Increased production of IL-17 by peripheral blood T cells has furthermore been detected in children with T1D [50]. In animal studies, a function for Th17 in T1D is supported by the observation that IL-17 is expressed in pancreas of NOD mice and that inhibition of IL-17 in this model leads to delayed onset of T1D during the effector phase of the disease [45]. Meanwhile, transfer of highly purified Th17 cells could cause diabetes in NOD/SCID recipients with similar rates of onset as in transfer of Th1 cells [51].

### Tregs

Tregs, suppressors of antigen-activated immune responses to self and non-self antigens, were first described in1975 [52]. Tregs play an indispensable role in maintaining immunological unresponsiveness and in suppressing excessive immune responses through cell contact-dependent mechanisms, by secretion of cytokines such as transforming growth factor (TGF)-β, IL-10 and IL-35 [53–55]. Transforming growth factor-β regulates multiple functions of T cell development, which plays a major role in T effector cells resistance to regulation and Tregs dysfunction [56]. Furthermore, the autocrine/paracrine TGF-β signalling in diabetogenic CD4^+^ T cells is essential for the control of T1D development [57]. In addition, IL-10 was believed to be a potent anti-inflammatory cytokine and ablation of IL-10 exacerbates autoimmune diseases, however, current evidence suggests that IL-10 deficiency does not accelerate T1D in NOD mice [58]. *In vitro* and *in vivo*, IL-35 has two well-known biological effects: suppression of the proliferation of T cells and the conversion of naïve T cells into a strongly suppressive induced Tregs, which has the capacity to protect β cells from autoimmune attack under certain circumstances [59,60].

Several markers for Tregs, such as human transcription factor forkhead box P3 (FoxP3), CTLA-4, CD25^high^ and CD127^low^, have been clarified [61]. Of these molecules, FoxP3 could be most essential for Tregs, which is not only for the development of Tregs, but also for the maintenance of their suppressive function [62]. It is now known that various subsets of Tregs exist in immune system including natural Tregs (nTregs), CD8^+^ Tregs, IL-10-producing type 1 Tregs and TGF-β-producing Th3 cells. In other classification, Tregs are divided into two subgroups, nTregs and inducible Tregs (iTregs) [63,64].

Adoptive transfer of Tregs has been shown to offer protection from T1D, whereas their experimental depletion or genetic deficiency in their numbers or activity promotes a more aggressive disease [56,65–68]. Furthermore, IL-2 administration has been shown to expand and activate Tregs in mice, while a short course of low-dose IL-2 administration at diabetes onset can reverse established disease [37,69,70]. Recent studies have also shown that T1D progression in NOD mice is associated with a decrease in numbers and function of Tregs in the inflamed islets, and defects in IL-2 production by effector T cells seem largely responsible [71].

In T1D patients, it was reported that there was increased apoptosis, and consequently, decreased viability and function of the Tregs in recent-onset T1D patients [72,73]. And autologous Tregs were a safe and well-tolerated therapy in children with T1D, which could inhibit or delay the destruction of pancreatic cells [74]. However, the study of Mikulkova*s proposed that the number of Tregs was no significant different in T1D compared with the normal group [75]. Recent reports also suggested there was no reduction in Tregs numbers in T1D and the main problem in T1D is extensively activated autoreactive T cells that are resistant to physiologically acting Tregs [74]. Collectively, these findings may support the view that the differences in earlier reports may be because of cross-identification of activated effector T cells by markers employed in identification of the Tregs population [56]. The functional defect rather than quantitative defect in the Tregs may be a more crucial factor in the development of T1D, which needs our further investigation.

## CD8^+^ T lymphocytes and T1D

CD8^+^ T cells, which recognize pathogen-derived peptides presented by major histocompatibility complex (MHC) class I molecules, were activated to proliferate and differentiate into cytotoxic T cell (CTL) and respond to infection by a number of intracellular bacteria [76,77]. In addition, effective CTL immunity is associated with long-term protection against chronic or subsequent exposure to the virus or tumour, through the stable induction of antigen-specific CD8^+^ T cell memory [78,79].

Previous studies have generally considered that both CD4^+^ and CD8^+^ T cells are involved in the pathogenesis of T1D and are thus capable of inducing β-cell death. However, pancreatic β cells express MHC class I, but lack MHC class II proteins, suggesting that direct cytotoxicity can only be mediated by CD8^+^ CTL that recognize peptide antigen: MHC class I complexes displayed on β cells [80–82]. For example, under histopathological examination CD8^+^ T cells were indeed found in the ‘insulitis’ of patients who died at onset of T1D, or in islets of monozygotic twins with recurrent T1D, after segmental pancreas transplantation from their non-diabetic co-twin [83]. Moreover, NOD mice deficient in MHC class I or MHC class I associated-β 2-microglobulin are protected from both insulitis and T1D, demonstrating that MHC class I presentation to CTL is necessary for disease initiation and progression to T1D [80].

Other studies have shown that IL-21 was required for efficient initial activation of autoreactive CD8^+^ T cells, which could rapidly kill β cells and therefore contribute to the development of T1D [84,85]. Key factors that can lead to β-cell death are cytotoxic CD8^+^ lymphocytes secreting perforin, direct action of cytokines such as IFN-γ, TNF-α and IL-1β, Fas–Fas-L interactions and nitric oxide synthesis [86]. Also noteworthy is the fact that, a population of CD8^+^ T cells recognizing an insulin-derived epitope (B:15–23) appears in the islets of NOD mice as early as 3 weeks of age [7]. The size of this population declines quickly with age and is replaced by other specificities, which targets a peptide from islet-specific glucose-6-phosphatase catalytic subunit-related protein (IGRP_206–214_) and are highly diabetogenic [7]. Furthermore, in the majority of T1D patients tested, there was a specific defect in CD8^+^ T cell recognition of HLA-E/Hsp60sp, which was associated with failure of self/non-self discrimination [87]. A failure of T-suppressor CD8^+^CD28^−^ T cell population was recognized in T1D [75].

## B lymphocytes in the pathogenesis of T1D

B lymphocytes and their products are not directly pathogenic to β cells, emerging evidence has revealed that they could promote autoimmunity by several mechanisms including: production of autoantibodies with consequent generation of immune complexes, antigen presentation to generate primary autoreactive T cell responses, contribution to the maintenance of CD4^+^T cell memory or production of pro-inflammatory cytokines [88,89].

Many studies have shown that autoantibodies are present in pre-diabetic and newly diagnosed patients with diabetes [90]. These include antibodies to proteins such as insulin, GAD, islet-cell antibodies, IAA, IA-2 and IA-2 β, which are also good markers for disease progression [91]. Moreover, a recent study demonstrated the necessity for B cells in the islets to promote survival of activated CD8^+^ T cells at the CTL transition stage, thereby accelerating disease progression [92]. On the other hand, B cells are crucial antigen-presenting cells in the initiation of T cell autoimmunity to islet β-cell autoantigens in T1D, although they do not present antigens as efficiently as dendritic cells. Migration of B cells into pancreatic lymph nodes in NOD mice is mediated predominantly by an α_4_β_7_ integrin/mucosal addressin cell adhesion molecule 1 pathway and partially by L-selectin/peripheral node addressin pathway and leucocyte adhesive protein-1 [93]. Furthermore, B cells could play other roles such as promoting normal lymphoid architecture and follicular dendritic cell formation [91]. Chronic depletion of B cells abrogates the destructive mononuclear cell infiltration of the pancreatic islets. B-cells depletion also exerted a similar protective effect and completely abrogated the development of insulitis in NOD mice [94]. Hu *et al*. recently demonstrated that combined treatment with intravenous anti-human CD20 and oral anti-CD3 reversed diabetes in >60% of mice newly diagnosed with diabetes, providing important pre-clinical evidence for the optimization of B cell-directed therapy for T1D [95]. In human, it has been reported that T1D developed in the absence of B cells, as seen in a patient who had X-linked agammaglobulinemia. This individual had very low serum levels of all classes of immunoglobulin and markedly decreased numbers of B cells in peripheral blood, but still developed T1D [96]. Taken together, B cells play an important role in disease development, especially in the animal models of T1D. Although it seems that B cells are not indispensable in human T1D, we would predict that B cells might assist the development of the T1D in other ways.

## NK cells in the pathogenesis of T1D

It is generally believed that NK cells are important players in innate immunity and are involved in direct killing of target cells that are transformed or infected by certain microorganisms without previous sensitization by recognizing class I HLA molecules on target cells through their membrane receptors [97,98].

Researchers have observed NK cells infiltrate islets of NOD mouse long ago, non-invasive islet inflammation is mainly mediated by NK cells [99,100]. Pancreatic NK cells, localized to the endocrine and exocrine parts, were present before T cells during disease development and did not require T cells for their infiltration [101]. The natural cytotoxicity receptors, which include NKp30, NKp44 and NKp46, are expressed almost exclusively on NK cells [102]. NKp46 is considered as the most specific NK cell marker, and the activating receptor NKp46 recognizes mouse and human ligands on pancreatic β cells leading to degranulation of NK cells [102]. NKp46-deficient mice had less development of T1D induced by injection of a low dose of streptozotocin [102]. The previous studies have shown a reduction in the frequency of NK cells in the peripheral blood in patients with T1D, and a reduced surface expression of the activating receptors NKp30 and NKp46 as well as lower mRNA levels of IFN-γ and perforin in NK cells of patients with long-standing T1D, when compared to controls without T1D [103,104]. In addition, NK cells express a wide range of both activating and inhibitory killer cell Ig-like receptor (KIRs), and the varied expression profile and balance of these receptors can dictate the NK cell function and activities [105]. Inhibitory KIRs may play an important role in immune regulation by actively promoting peripheral tolerance, enhancing effector cell survival or dampening immune responses [105]. However, activating KIRs are implicated in conditions including active host defence against infectious organisms. Normal T cells express very few or no KIRs, but KIR expression can be detected on a small subset of T cells in patients with T1D [105]. Furthermore, NK cells exert cytolitic activity and secrete cytokines and chemokines like IFN-γ, TNF-α and GM-CSF, the immunoregulatory cytokines IL-5, IL-10, IL-13 and the chemokines MIP-1α [4]. NK cells were also detected rarely in inflamed islets in pancreas samples of human, which suggested that NK cells participate in the initial pro-inflammatory process, but may become hyporesponsive because of exhaustion or regulation in later stage of T1D [101,106].

## APC in the pathogenesis of T1D

Antigen-presenting cells play a crucial role in T cell differentiation by providing co-stimulatory signals and cytokines at T cell priming [42]. Dendritic cells (DCs), a major subset of APCs, are widely distributed throughout non-lymphoid and lymphoid tissues though low in number [107]. Dendritic cells comprise two major classes: plasmacytoid DCs (pDCs) and conventional or classical DCs (cDCs) [108]. Dendritic cells recognize pathogens using pattern recognition receptors, including Toll-like receptors (TLRs), then they migrate to T cell areas of lymphoid organs and produce cytokines such as IL-1, TNF-α as well as IL-12 family [108,109]. In addition, DCs are uniquely capable of stimulating clonal expansion of naïve T cells and in the modulation of their development into autoreactive Th1 lymphocytes or immunosuppressive Th2 cells critical for maintenance of immunological homoeostasis [13,107].

In T1D, the increased accumulation of DCs along with Mφ in the earliest islet infiltrates of both humans with T1D and NOD mice suggests an important role for these cells in the pathogenesis of T1D [97]. DCs are responsible for the presentation of islet-cell derived antigens to diabetogenic T cells as well as to regulatory T cell populations within the pancreas and pancreatic lymph node [41]. Results from both NOD mice and patients with T1D document abnormalities in DCs function such as increased NF-κB activity, decreased expression of indoleamine-2,3-dioxygenase, and altered costimulatory and cytokine secretion profiles [41,110–113]. It is now admitted that DCs play a major role in Tregs control, particularly pDCs were able to induce potent proliferation of Tregs in the absence of exogenous IL-2 and down-regulate their suppressive activity *in vitro* [114,115]. In the context of T1D, cDCs can induce the expansion of self-antigen-specific Tregs that are key players in the prevention of T1D and are promising therapeutic targets in this disease [116,117]. A pathogenic role of pDCs in T1D is supported by observations in both humans and rodent models that type 1 IFN is produced in pancreatic islets and it could induce or promote the development of the disease [116]. Meyers *et al*. have further reported that pDCs and cDCs from new-onset patients may have altered TLR7/8 and TLR4 signalling, respectively, with increased pro-inflammatory cytokine and chemokine expression levels in sera [118]. A significantly higher proportion of T1D patients have ‘very low suppression’ activity by autologous Tregs compared to controls, which may be because of defects in APC [119].

Cytokines secreted by DCs are considered as critical mediators of the T1D as well. For example, IL-1 signalling has roles in β-cell dysfunction and destruction *via* the NF-κB and mitogen-activated-protein-kinase pathways, leading to endoplasmic reticulum and mitochondrial stress and eventually activating the apoptotic machinery [120]. IL-1 can also acts on T lymphocyte regulation [120]. Genetic or pharmacological abrogation of IL-1 action reduces disease incidence in animal models of T1D [120]. On the other hand, IL receptor antagonist (IL1-RA) correlates positively with residual β-cell function by limiting aggressive or inflammatory immune reactivity [121]. As another example, TNF-α plays an important role in the initiation of T1D by regulating the maturation of DCs and the activation of islet-specific pancreatic lymph node T cells [122].

In conclusion, DCs seem to have an essential role in the pathogenesis of T1D, however, the potential role of DCs in the therapy of T1D needs further study.

## Other innate immune cells

In addition, other innate immune cells such as γδT, NKT and macrophages play essential roles in the pathogenesis of TIDM. γδT cells protected NOD mice from diabetes in a TGF-β-dependent manner [123]. Natural killer T cells are divided into three subsets: type I, or invariant NK (iNK) T, type II NK T and NK T-like cells [124]. Numerical and functional deficiencies in iNK T cells develop in islets during progression to T1D in NOD mice, and T1D can be prevented in NOD mice by increasing iNKT cell numbers or by specific iNKT cell stimulation [125,126]. A number of studies have confirmed that TLR-mediated innate immune responses could contribute to the induction of diabetes in mice [127]. For example, apoptotic β cells can activate antigen-presenting innate immune cells *via* TLR2, which subsequently prime islet-specific diabetogenic CD4^+^ T cells in NOD mice; mouse and human islet cells express TLRs and their trigger increases the secretion of pro-inflammatory chemokines such as CXCL-10, which is able to attract T cells, macrophages and dendritic cells into the pancreatic islets; viral infections may play an important role in T1D, some studies have demonstrated that double-stranded RNA (dsRNA) of most viruses could induce pancreatic β-cell apoptosis by activation of the TLR3 on pancreatic β cells in animal models and in primary pancreatic β cells [4].

## Conclusion

In this review, we have summarized the latest evidence on the cellular immunological mechanism of T1D, which is caused by many immune cells and cytokines. Both CD4^+^ and CD8^+^ T lymphocyte have been implicated as key players in β-cell destruction, while B cells might assist the development of the T1D by several indirect mechanisms. In addition, NK cells, DCs and other innate immune cells also take part in the damage of pancreatic β cells *via* some uncertain mechanisms, which ultimately lead to the eventual destruction of β cells.

There is increasing experimental data and emerging evidence from clinical trials to treat T1D though most have proved too toxic or have failed to provide long-term β cell protection [128]. In early efforts to block the autoimmune process and preserve β cell functions in newly diagnosed T1D patients, immunosuppressive agents, such as azathioprine, cyclophosphamide and cyclosporine were introduced [39,129]. Current treatments of the T1D are mainly based on man-made insulin and pancreatic-islet transplantation. Unfortunately, although these treatments have reduced mortality and significantly lengthened patients* life expectancies, the major problem is that they have no effect on the autoimmune process with numerous adverse effects [130]. Recent progress has improved our understanding of the immune therapy in T1D, which is approached at different stages as follows: primary prevention is treatment of individuals at increased genetic risk; secondary prevention involving non-autoantigen-specific therapies or autoantigen-specific therapies is targeted at individuals with persistent islet autoantibodies; tertiary prevention includes non-autoantigen-specific approaches and autoantigen-specific therapies [131]. Anti-CD3 mAbs mitigates the deterioration in insulin production and improves metabolic control, which appears to be the most effective therapeutic strategy until now [132]. It has also been indicated that haematopoietic stem cell transplantation for the treatment of autoimmunity is possible to provide protection from disease onset, as well as reverse the autoimmune state in T1D [133]. Similarly, mesenchymal stem cells (MSCs) have emerged as a potential new therapy for T1D. Several studies from the past few years show that MSCs can minimize β-cell damage by providing survival signals and simultaneously modulate the immune response by inhibiting activation, and proliferation of several immune cell types [134,135]. Furthermore, it has been demonstrated that γ-aminobutyric acid in islet β cell could activate phosphatidylinositol 3-kinase/protein kinase B (PI3-K/Akt)–dependent growth and survival pathways, which provides a potential therapy to preserve β-cell mass and prevent the development of T1D [136]. Intriguingly, new research have proven that inhibition of the PI3-K γ pathway by AS605240 could efficiently prevent and reverse diabetes in T1D [127,137]. And Tregs may be further exploited for the treatment and prevention of T1D, which are promising cells in maintaining immunological unresponsiveness and in suppressing excessive immune responses. Therefore, future studies will be directed at searching for an effective, safe and long-lasting strategy to enhance the regulation of a diabetogenic immune system with limited toxicity and without global immunosuppression [39].
